# The association of functional polymorphisms in genes expressed in endothelial cells and smooth muscle cells with the myocardial infarction

**DOI:** 10.1186/s40246-018-0189-8

**Published:** 2019-01-24

**Authors:** Yilan Li, Shipeng Wang, Dandan Zhang, Xueming Xu, Bo Yu, Yao Zhang

**Affiliations:** 1 Department of Cardiology, the 2nd Affiliated Hospital of Harbin Medical University, Harbin, 150001 China; 2 Key Laboratory of Myocardial Ischemia, Ministry of Education, Harbin Medical University, Harbin, 150001 China

**Keywords:** PECAM1, HIF1A, KIAA1462, Single-nucleotide polymorphism, Myocardial infarction, Coronary artery disease

## Abstract

**Background:**

The association of platelet endothelial cell adhesion molecule 1 (PECAM1), hypoxia-inducible factor 1 subunit alpha (HIF1A), and KIAA1462 in myocardial infarction (MI) was investigated. The study included 401 Han Chinese MI patients and 409 controls. Three tag single-nucleotide polymorphisms (SNPs)—PECAM1 rs1867624, HIF1A rs2057482, and KIAA1462 rs3739998—were selected. SNP genotyping was performed by an improved multiplex ligation detection reaction assay. A systematic review and meta-analysis of studies including 3314 cases and 2687 controls on the association of 5 HIF1A SNPs and the overall risk of MI or coronary artery disease (CAD) was performed.

**Results:**

The rs1867624 variants were associated with high TG concentrations (*p* = 0.040) and the rs2057482 variants were associated with decreased HDL-C in MI patients compared with the control group (*p* = 0.003). Rs2057482 SNP interacted with age to influence TC levels. The SNP of rs3739998 interacted with sex and hypertension to modulate CRE and TG levels, respectively (*p* < 3.04E-5-0.002). No association between the three SNPs and susceptibility to MI was found (*p* > 0.05 for all). In the meta-analysis of HIF1A, the rs11549465 C > T and rs10873142 T > C polymorphisms, but not rs2057482, rs11549467, and rs41508050, were correlated with overall MI or CAD risk.

**Conclusions:**

Taken together, this study provides additional evidence that genetic variation of the PECAM1 rs1867624 and HIF1A rs2057482 can mediate lipid levels in MI patients.

**Electronic supplementary material:**

The online version of this article (10.1186/s40246-018-0189-8) contains supplementary material, which is available to authorized users.

## Background

Coronary artery disease (CAD) is a complex, multifactorial, and polygenic disorder caused by an excessive inflammatory response to various forms of injury, resulting in endothelial dysfunction in the arterial walls leading to accelerated atherosclerosis [1]. In the blood vessel wall, the basement membrane underlies the endothelium and surrounds smooth muscle cells (SMCs) [2]. The basement membrane not only serves as an extracellular scaffold but also regulates cell behavior [3]. Abnormalities of vascular endothelial cells (ECs) and SMCs play important roles in the pathogenesis of atherosclerosis, the vascular pathology underlying CAD [4].

The recent targeted gene array derived from earlier genome-wide association studies (GWAS) results and performed meta-analysis of results have made huge advances in identifying genetic components of CAD risk with 15 mainly novel risk loci, including single-nucleotide polymorphisms (SNPs) in or near genes involved in major lipids, blood pressure, and vascular smooth muscle cell differentiation [5]. Several of these have been validated by large-scale association studies [6, 7]. However, the functional relationship between many of these loci and the risk of coronary heart disease (CAD)/myocardial infarction (MI) remains to be determined [8]. Three of the new CAD-associated regions identified in the current analysis include genes that encode proteins expressed in smooth muscle cells (PECAM1, HIF1A) and endothelial cells (KIAA1462).

Rs1867624 is upstream of PECAM1, which encodes platelet endothelial cell adhesion molecule 1, a cell adhesion protein that play a role in endothelial cell sprouting [9]. It helps leukocytes adhere to the endothelium and migrate into the intima layer of the artery [10]. Reschner et al. have demonstrated the association between PECAM1*L/L genotype and MI in Slovenian population [11]. Serebruany at al. determined plasma levels of PECAM-1 and prospectively compare these data with the discharge diagnosis in patients presenting with chest pain in a community hospital emergency department [12]. Levels of soluble PECAM-1 were increased in patients with AMI. Wenzel et al. investigated two polymorphisms (Leu125Val and Ser563Asn) in 98 patients with coronary artery stenoses and 103 healthy controls [13]. Both the variants were more frequent in patients as was the homozygous combination Val125/Asn563 (0.43 vs 0.2). Together, these findings prioritize PECAM1 as a candidate causal gene for this CAD-associated region in humans.

HIF1A is a transcriptional factor encoded by the HIF1A gene located on chromosome 14 (14q23.2) and plays a critical role in the regulation of different cellular processes involved in the preservation of oxygen homeostasis [1]. Imanishi et al. show that hypoxia-inducible factor-1α (Hif-1α) expressed in smooth muscle cells is involved in angiotensin II (Ang II)-induced vascular remodeling in an in vivo model [14]. Alberto et al. suggested that rs2057482 polymorphism is involved in the risk of developing CAD and is associated with some metabolic parameters and cardiovascular risk factors. The rs2057482 T allele was associated with decreased risk of obesity, central obesity, hypertension, hypercholesterolemia, hypertriglyceridemia, and increased risk of T2DM [1]. Guo et al. showed that the genetic mutations within HIF-1A (rs2057482) could alter susceptibility to perimenopausal CAD [15].

KIAA1462 is newly identified locus, which is associated with coronary artery disease, the KIAA1462 protein participates in the regulation of adherens junctions and cytoskeleton formation in endothelial cells [16]. Thus, it is involved in the support of integrity and permeability of the endothelium, which is always exposed to the mechanical action of blood flow. In turn, the endothelium permeability affects the migration rate of monocytes, which morph into macrophages that favor the formation and development of atherosclerotic plaques [17]. The G allele at rs3739998 leads to a missense variant (Ser1002Thr) which is located in exon 3 of the KIAA1462 gene [18]. Akashi et al. also independently identified junctional protein associated with coronary artery disease (JCAD), which is a gene product of KIAA1462 through localization-based expression cloning of novel junctional proteins [19].

In light of previous studies, we selected three genes expressed in smooth muscle cells and one endothelial-related gene and aimed to test their contribution to the risk of MI in Han Chinese.

## Results

### Population characteristics

The clinical characteristics of the 810 study participants are shown in Table 1. The majority (82.0%) were men (60.3%) were current smokers, and 26.2% drank alcohol. The hypercholesterolemia and triglycerides levels were not different between controls and MI patients (*p* > 0.05 for all). The MI patients had higher smoking, alcohol, diabetes, hypertension, WBC, FBG, CRE, AST, total cholesterol, but lower HDL cholesterol and LDL cholesterol.

**Table 1:** Baseline characteristics of the individuals included in the study

Variables	MI (*n* = 401)	Controls (*n* = 409)	OR (95% CI)^a^	*p* value^b^
Age, years	58.20 (11.65)	56.34 (9.52)	0.84 (0.75-1.24)	0.223
Male sex, No. (%)	329 (82.0%)	339 (82.9%)	0.94 (0.66- 1.36)	0.753
Smoking, No. (%)	242 (60.3%)	187 (45.7%)	*0*.*56* (*0.37*-*0*.*72)*	*3*.*04E*-*05*^c^
Alcohol, No. (%)	105 (26.2%)	82 (20.0%)	*0*.*62* (*0*.*39*-*0*.*82*)	*0*.*038*
Diabetes, No. (%)	99 (26.7%)	50 (12.2%)	*0*.*43* (*0*.*29*-*0*.*62*)	*4*.*71E*-*06*
Hypertension, No. (%)	192 (47.9%)	143 (35.0%)	*0*.*59* (*0*.*44*-*0*.*78*)	*1*.*90E*-*4*
Hypercholesterolemia, No. (%)	55 (13.7%)	39 (9.5%)	0.66 (0.43-1.03)	0.063
WBC, 10^9^/L	11.96 (3.81)	6.84 (1.90)	*1*.*93* (*1*.*76*-*2*.*12*)	*1*.*78E*-*80*
FBG, mmol/L	6.44 (5.10, 8.88)	5.34 (4.93, 6.04)	*1*.*15* (*1*.*09*-*1*.*21*)	*1*.*25E*-*7*
CRE, μmol/L	81.00 (69.00, 95.30)	71.00 (62.00, 80.00)	*1*.*04* (*1*.*03*-*1*.*05*)	*3*.*22E*-*18*
AST, U/L	37.00 (22.00, 117.50)	24.00 (20.00, 28.00)	*1*.*04* (*1*.*03*-*1*.*05*)	*2*.*54E*-*32*
Total cholesterol, mmol/L	4.56 (1.05)	4.96 (0.84)	*0*.*65* (*0*.*56*-*0*.*76*)	*7*.*03E*-*9*
Triglycerides, mmol/L	1.38 (0.95,1.93)	1.32 (0.92, 2.02)	0.99 (0.88-1.11)	0.764
HDL cholesterol, mmol/L	1.23 (1.06, 1.43)	1.31 (1.13, 1.52)	*0*.*50* (*0*.*33*-*0*.*75*)	*1*.*65E*-*3*
LDL cholesterol, mmol/L	2.77 (0.83)	3.30 (0.73)	*0*.*44* (*0*.*36*-*0*.*53)*	*1*.*92E*-*20*

Values are mean ± SD or *n* (%), continuous variables that have skewed distribution are expressed as median (25, 75 percentiles)

Type 2 diabetes was diagnosed: (1) fasting plasma glucose (FPG) ≥ 7.0 mmol/L; (2) 2 h postprandial glucose ≥ 11.1 mmol/L; or (3) use of anti-diabetic medications. Hypertension was defined as systolic/diastolic blood pressure ≥ 140 mmHg or ≥ 90 mmHg or use of antihypertensive medications. Hypercholesterolemia was defined as use of cholesterol-lowering medications or total serum cholesterol > 200 mg/dl. *WBC* white blood cell, *FBG* fasting blood glucose, *AST* aspartate transaminase, *CRE* creatinine, *HDL* high-density lipoprotein, *LDL* low-density lipoprotein

^a^Binary regression

^b^Two-sided chi-square test or independent-samples 푡 test

^c^*p* values under 0.05 were shown in italic font

### Genotype and allele frequencies in patients and controls

The genotype and allele frequencies of three SNPs selected for study are shown in Table 2. The selected SNPs all displayed a MAF > 0.05 and the allele frequencies were similar to observations derived from the pooled sample, according to 1000 Genomes (rs1867624, *C* = 0.2979; rs2057482, *T* = 0.2424; rs3739998, *G* = 0.2518.). No deviations from Hardy–Weinberg equilibrium were observed in either cases or controls. The genotype and allele frequencies of the rs1867624, rs2057482, and rs3739998 SNPs in MI patients and controls were not significantly different (all *p* > 0.05).

**Table 2:** Genotypic and allelic frequencies of three SNPs in MI cases and controls

SNP/group	Genotype^a^ (*n* (%))			χ^2^	*p*	Allele (*n* (%))		χ^2^	*p*	OR (95% CI)
rs1867624	CC	CT	TT			C	T			
Case	28 (0.07)	133 (0.33)	240 (0.60)			189 (0.24)	613 (0.76)	2.94	0.086	1.23 (0.97–1.23)
Control	18 (0.04)	128 (0.31)	263 (0.64)	0.88	0.349	164 (0.20)	654 (0.80)			
rs2057482	TT	CT	CC			T	C			
Case	17 (0.04)	132 (0.33)	252 (0.63)			166 (0.21)	636 (0.79)	0.11	0.75	1.04 (0.82–1.33)
Control	22 (0.05)	120 (0.29)	267 (0.65)	2.94	0.09	164 (0.20)	654 (0.80)			
rs3739998	GG	GC	CC			G	C			
Case	20 (0.05)	132 (0.33)	249 (0.62)			172 (0.21)	630 (0.79)	0.26	0.611	0.94 (0.74–1.19)
Control	24 (0.06)	136 (0.33)	249 (0.61)	0.23	0.630	184 (0.22)	634 (0.78)			

^a^All are in HWE. *SNP* Single nucleotide polymorphism, *MI* myocardial infarction

### Genotypes of the three SNPs and the risk of MI

Results of the genetic model analysis are shown in Table 3. Comparison of both heterozygous and homozygous carriers of the minor allele (C) with homozygous carriers of the major allele (T), suggesting a dominant genetic effect, revealed that the PECAM1 rs1867624 SNPs were not associated with MI. No association of rs2057482 and rs3739998 and MI were observed. Similar, but weaker trends were observed for the recessive model, with no significant associations of the three SNPs with MI (all *p* > 0.05).

**Table 3:** Genetic models analyses of the association between three SNPs and MI susceptibility

SNP/group	Genotype		χ^2^	*p*	OR (95% CI)
rs1867624					
Dominant	TT + CT	CC			
Case	161 (0.40)	240 (0.60)	1.71	0.192	1.21 (0.91–1.61)
Control	146 (0.36)	263 (0.64)			
Recessive	TT	CT + CC			
Case	28 (0.07)	373 (0.93)	2.52	0.112	1.63 (0.89–2.98)
Control	18 (0.04)	391 (0.96)			
rs2057482					
Dominant	TT + CT	CC			
Case	149 (0.37)	252 (0.63)	0.52	0.47	1.11 (0.83–1.48)
Control	142 (0.35)	267 (0.65)			
Recessive	TT	CT + CC			
Case	17 (0.04)	384 (0.96)	0.57	0.45	0.78 (0.41–1.49)
Control	22 (0.05)	387 (0.95)			
rs3739998					
Dominant	CC + GC	GG			
Case	152 (0.38)	249 (0.62)	0.13	0.722	0.95 (0.71–1.26)
Control	160 (0.39)	249 (0.61)			
Recessive	CC	GC + GG			
Case	20 (0.05)	381 (0.95)	0.31	0.580	0.84 (0.46–1.55)
Control	24 (0.06)	385 (0.94)			

*SNP* single nucleotide polymorphism, *MI* myocardial infarction

### Genotype and lipid levels

We expected that genetic risk associated with the SNPs would be reflected by established CAD risks, including total cholesterol (TC), triglycerides (TGs), high-density lipoprotein cholesterol (HDL-C), low-density lipoprotein cholesterol (LDL-C), creatinine (CRE), or fasting blood glucose (FBG). As shown in Table 4, the rs1867624 variants were associated with high TG concentrations (*p* = 0.040) and the rs2057482 variants were associated with decreased HDL-C in MI patients compared with the control group (*p* = 0.003). None of the three SNPs were associated with TC, LDL-C, CRE, or FBG in MI patients (*p* > 0.05).

**Table 4:** Lipid levels according to genotype in cases and controls

SNP	Genotype (counts)	TC mmol/L	TG mmol/L	HDL-C mmol/L	LDL-C mmol/L	CRE μmol/L	FBG mmol/L
rs1867624							
Case	CC (28)	4.47 ± 1.18	1.32 ± 0.95	1.39 ± 0.62	2.80 ± 0.90	83.03 ± 13.66	6.38 ± 4.37
	CT (133)	4.53 ± 1.09	1.71 ± 1.23	1.29 ± 0.52	2.72 ± 0.90	84.04 ± 32.32	7.44 ± 3.58
	TT (240)	4.59 ± 1.00	1.65 ± 1.08	1.25 ± 0.33	2.79 ± 0.79	86.88 ± 30.36	7.05 ± 3.94
	*p*	0.876	*0*.*040*	0.744	0.737	0.659	0.703
Control	CC (18)	5.13 ± 1.07	1.80 ± 1.87	1.42 ± 0.43	3.30 ± 0.74	72.83 ± 12.28	6.95 ± 3.96
	CT (128)	5.04 ± 0.78	1.76 ± 1.29	1.36 ± 0.36	3.34 ± 0.74	71.35 ± 12.20	5.81 ± 1.91
	TT (263)	4.89 ± 0.86	1.61 ± 1.14	1.38 ± 0.36	3.25 ± 0.75	70.46 ± 13.89	5.93 ± 1.69
	*p*	0.262	0.353	0.843	0.396	0.471	0.072
rs2057482							
Case	TT (17)	4.92 ± 1.07	1.48 ± 0.75	1.40 ± 0.22	3.10 ± 0.92	83.88 ± 24.60	8.22 ± 3.64
	CT (132)	4.55 ± 0.98	1.59 ± 0.82	1.20 ± 0.24	2.83 ± 0.84	88.12 ± 31.46	7.69 ± 3.60
	CC (252)	4.54 ± 1.08	1.69 ± 1.28	1.32 ± 0.49	2.71 ± 0.82	85.90 ± 28.11	7.61 ± 3.37
	*p*	0.493	0.811	*0*.*003*	0.203	0.944	0.424
Control	TT (22)	4.96 ± 0.60	1.80 ± 1.73	1.43 ± 0.33	3.12 ± 0.65	72.00 ± 11.69	5.98 ± 2.16
	CT (120)	5.01 ± 0.92	1.77 ± 1.20	1.40 ± 0.41	3.32 ± 0.84	71.18 ± 13.94	5.94 ± 1.87
	CC (267)	4.92 ± 0.83	1.61 ± 1.19	1.36 ± 0.35	3.28 ± 0.71	70.60 ± 13.16	5.93 ± 1.93
	*p*	0.327	0.064	0.396	0.375	0.910	0.819
rs3739998							
Case	GG (20)	4.47 ± 1.22	1.78 ± 1.64	1.20 ± 0.34	2.61 ± 1.02	92.34 ± 40.47	8.49 ± 4.37
	GC (132)	4.55 ± 1.04	1.64 ± 1.26	1.27 ± 0.39	2.79 ± 0.85	83.00 ± 28.88	7.24 ± 4.06
	CC (249)	4.58 ± 1.04	1.64 ± 1.00	1.29 ± 0.45	2.76 ± 0.81	86.55 ± 29.90	6.97 ± 3.70
	*p*	0.866	0.371	0.855	0.927	0.364	0.335
Control	GG (24)	4.78 ± 0.67	1.71 ± 1.38	1.37 ± 0.28	3.09 ± 0.61	71.54 ± 9.31	6.06 ± 1.83
	GC (136)	4.98 ± 0.83	1.62 ± 1.31	1.37 ± 0.32	3.34 ± 0.72	70.68 ± 13.46	5.87 ± 1.84
	CC (249)	4.94 ± 0.87	1.68 ± 1.17	1.37 ± 0.39	3.26 ± 0.76	70.87 ± 13.57	5.96 ± 1.97
	*p*	0.594	0.327	0.724	0.352	0.949	0.641

*SNP* single nucleotide polymorphism, *TC* total cholesterol, *TG* triglyceride, *HDL*-*C* high-density lipoprotein cholesterol, *LDL*-*C* low density lipoprotein cholesterol, *CRE* creatinine, *FBG* fasting blood glucose. The value was presented as mean ± SD and significance (*p* < 0.05) was determined by the Kruskal–Wallis test

### Interactions of the three SNPs and drinking, smoking, age, sex, and hypertension on lipid levels and the risk of MI

The interactions of the three SNPs and drinking, smoking, age, sex, and hypertension on lipid levels and the risk of MI are shown in Table 5. Rs2057482 SNP interacted with age to influence TC levels. The SNP of rs3739998 interacted with sex and hypertension to modulate CRE and TG levels, respectively.

**Table 5:** The *p* values for interactions of genotypes and age, drinking and smoking, on lipid levels and the risk of CHD

SNP	Factor	TC	TG	HDL-C	LDL-C	CRE	FBG
rs1867624	Drinking	0.140	0.391	0.778	0.037	0.528	0.642
	Smoking	0.957	0.470	0.936	0.485	0.684	0.667
	Age	0.010	0.041	0.930	0.075	0.020	0.756
	Sex	0.096	0.005	0.483	0.656	0.028	0.005
	Hypertension	0.567	0.003	0.270	0.284	0.738	0.311
rs2057482	Drinking	0.807	0.808	0.388	0.508	0.303	0.560
	Smoking	0.945	0.241	0.346	0.482	0.921	0.394
	Age	*0*.*001*	0.229	0.640	0.175	0.329	0.621
	Sex	0.265	0.019	0.234	0.687	0.186	0.032
	Hypertension	0.009	0.207	0.194	0.244	0.278	0.121
rs3739998	Drinking	0.484	0.331	0.559	0.880	0.900	0.111
	Smoking	0.669	0.040	0.555	0.356	0.906	0.175
	Age	0.004	0.002	0.115	0.055	0.779	0.576
	Sex	0.492	0.344	0.014	0.904	*3*.*04E*-*5*	0.131
	Hypertension	0.102	*0*.*002*	0.403	0.419	0.775	0.087

*SNP* single nucleotide polymorphism, *TC* total cholesterol, *TG* triglyceride, *HDL*-*C* high-density lipoprotein cholesterol, *LDL*-*C* low-density lipoprotein cholesterol, *FBG* fasting blood glucose, *CRE* creatinine, *MI* myocardial infarction. A *p* < 0.003 was considered statistically significant after Bonferroni correction

### Systematic meta-analysis of HIF1A with MI or CAD

**Fig. 1:**
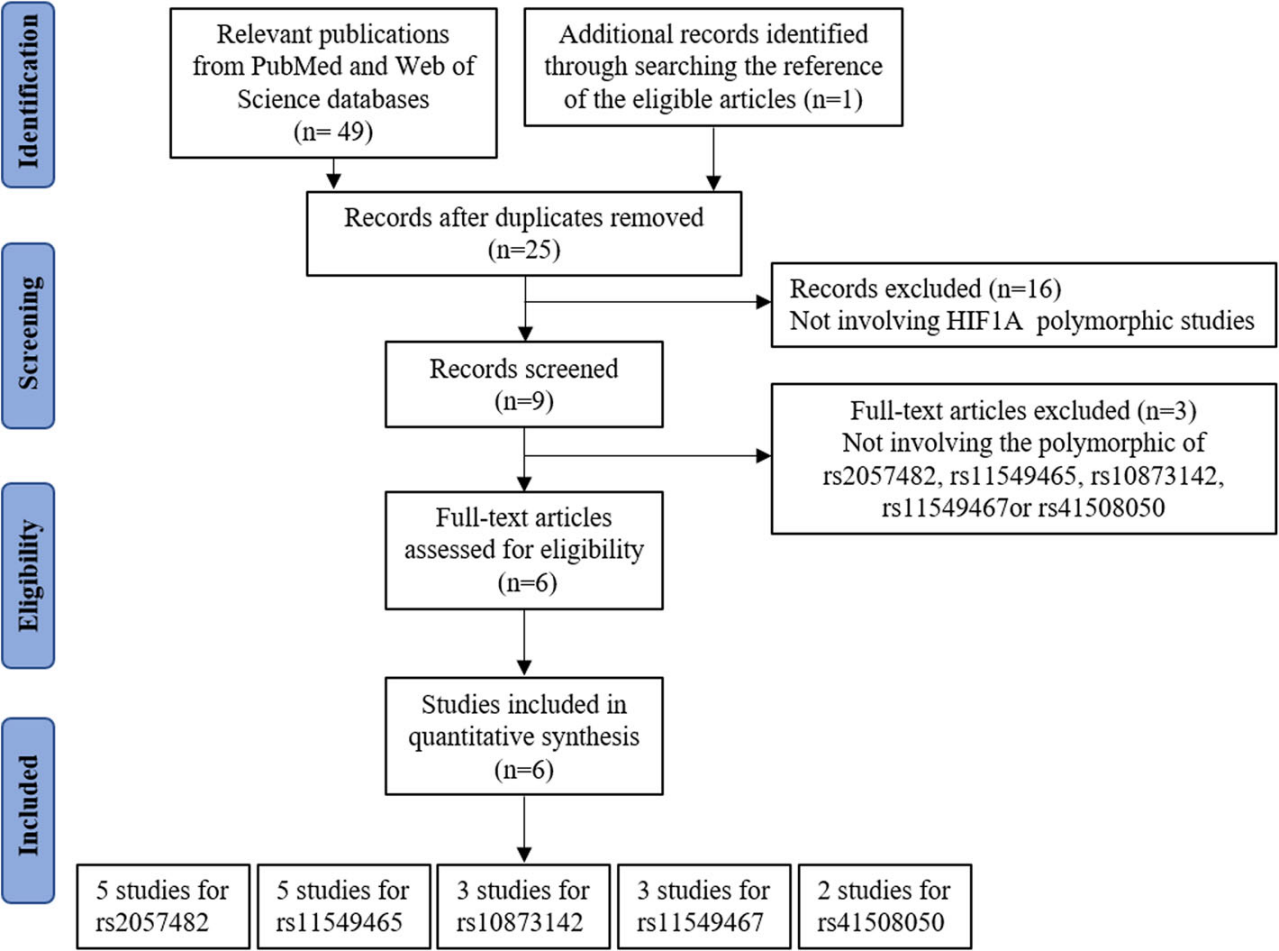
Flow diagram for the process of study selection

Recent studies have shown that the SNP rs2057482 in the HIF1A is associated with susceptibility to CAD [1]. However, our studies have shown inconsistent results. Therefore, a systematic meta-analysis was carried out to evaluate the association between HIF1A and MI or CAD in the literature. Following the application of strict screening criteria, 6 articles evaluating a total of 3314 cases and 2687 controls concerning MI or CAD were ultimately included in our quantitative analysis (Fig. 1). The general characteristics of the included studies are listed in Table 6.

**Table 6:** Characteristics of included studies on HIF1A polymorphisms and MI or CAD risk included in the meta-analysis

Cite	First author	Year	Ethnicity	Country/Region	Case	Control	Genotype distribution						Genotyping methods	Disease	*p* for HWE^a^
							Case		Control						
							rs2057482 C > T								
							CC	CT	TT	CC	CT	TT			
Caucasian															
[28]	Hlatky	2007	Caucasian	California	909	466	641	239	29	309	134	23	TaqMan	AMI	0.09
[1]	Lopez-Reyes	2014	Caucasian	Mexico	949	676	809	134	6	547	120	9	TaqMan	CAD	0.41
[29]	Duran	2015	Caucasian	Spain	497	111	339	148	10	70	38	3	TaqMan	CAD	0.42
Asian															
[30]	Zheng	2009	Asian	Korea	14	361	8	5	1	233	117	11	SNaPShot	AMI	0.42
[15]	Guo	2017	Asian	China	589	860	476	93	20	726	125	9	TaqMan	CAD	0.17
							rs11549465 C > T								
							CC	CT	TT	CC	CT	TT			
[28]	Hlatky	2007	Caucasian	California	909	466	747	151	11	358	97	11	TaqMan	AMI	0.16
[30]	Zheng	2009	Asian	Korea	14	360	13	1	0	325	33	2	SNaPShot	AMI	0.26
[31]	Liu	2013	Asian	China	356	213	346	10	0	208	5	0	PCR	CAD	0.86
[29]	Duran	2015	Caucasian	Spain	518	112	402	111	5	84	26	2	TaqMan	CAD	0.99
[15]	Guo	2017	Asian	China	589	860	324	10	255	449	11	400	TaqMan	CAD	*0.00*
							rs10873142 T > C								
							TT	TC	CC	TT	TC	CC			
[28]	Hlatky	2007	Caucasian	California	909	466	549	305	55	249	174	43	TaqMan	AMI	0.12
[30]	Zheng	2009	Asian	Korea	14	360	7	6	1	199	141	20	SNaPShot	AMI	0.44
[15]	Guo	2017	Asian	China	609	860	355	209	45	442	347	71	TaqMan	CAD	0.80
							rs11549467 G > A								
							GG	GA	AA	GG	GA	AA			
[28]	Hlatky	2007	Caucasian	California	909	466	900	9	0	456	10	0	TaqMan	AMI	0.81
[31]	Liu	2013	Asian	China	356	213	340	16	0	204	9	0	PCR	CAD	0.75
[15]	Guo	2017	Asian	China	589	860	326	17	246	454	19	387	TaqMan	CAD	*0.00*
							rs41508050 C > T								
							CC	CT	TT	CC	CT	TT			
[28]	Hlatky	2007	Caucasian	California	909	466	903	6	0	457	9	0	TaqMan	AMI	0.83
[15]	Guo	2017	Asian	China	589	860	581	8	0	843	17	0	TaqMan	CAD	0.77

^a^HWE in control; *AMI* acute myocardial infarction, *CAD* coronary artery disease

*p* values under 0.05 where shown in italic font

**Fig. 2:**
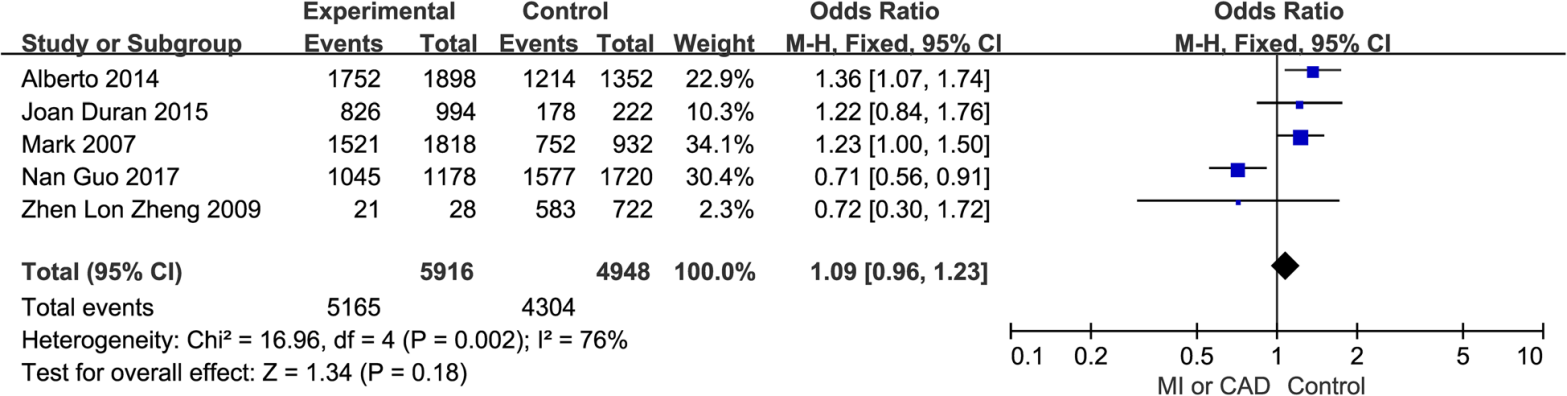
OR and 95% CIs of the associations between HIF1A rs2057482 CT polymorphism and MI or CAD in allele model. The squares and horizontal lines correspond to the study-specific OR and 95% CI. The area of the squares indicates the study-specific weight (inverse of the variance). The diamond represents the pooled OR and 95% CI

The association between the HIF1A rs2057482 C > T polymorphism and MI or CAD risk was examined in 5 relevant studies involving 2958 patients and 2474 healthy controls. No significant overall associations were identified in allele genetic model (OR = 1.09, 95% CI = 0.96–1.23, *p* = 0.18, *I*^2^ = 76%) (Fig. 2). The sensitivity analysis, which was conducted by omitting studies one by one to examine the stability of the pooled ORs, revealed an obvious change when data from Nan Guo’s study was removed (OR = 1.25, 95% CI = 1.09–1.44, *p* = 0.002, *I*^2^ = 0%) [15] (Additional file 1: Figure S1). Compared with other studies in the meta-analysis, the frequency of the T allele in Korea was greater than 25% and lower than 17% in Caucasians. We perform a separate meta-analysis for Asians and Caucasians since minor allele frequency is quite different among populations. For the Caucasians populations, the HIF1A rs2057482 C > T polymorphism was associated with CAD (OR = 1.27, 95% CI = 1.10–1.47, *p* = 0.001, *I*^2^ = 0%) (Additional file 1: Figure S2). For the Asians populations, the HIF1A rs2057482 C > T polymorphism was associated with CAD (OR = 0.71, 95% CI = 0.56–0.91, *p* = 0.006, *I*^2^ = 0%) (Additional file 1: Figure S3).

**Fig. 3:**
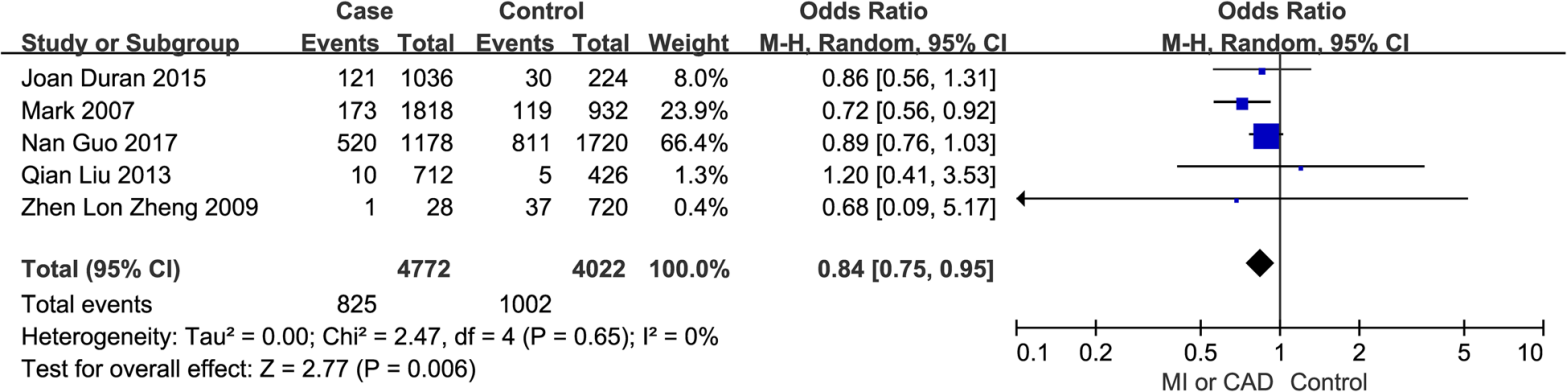
OR and 95% CIs of the associations between HIF1A rs11549465 CT polymorphism and MI or CAD in allele model. The squares and horizontal lines correspond to the study-specific OR and 95% CI. The area of the squares indicates the study-specific weight (inverse of the variance). The diamond represents the pooled OR and 95% CI

The association between HIF1A rs11549465 C > T polymorphism and CAD risk was examined in 5 studies involving 2011 cases and 2386 controls. A protective effect on CAD development was observed in the variant T allele (OR = 0.84, 95% CI = 0.75–0.95, *p* = 0.006, *I*^2^ = 0%) (Fig. 3).

For the rs10873142 T > C polymorphism, the allele model was associated with increased overall risk of CAD (OR = 1.28, 95% CI = 1.14–1.45, *p* < 0.0001, *I*^2^ = 0%) (Additional file 1: Figure S4). The rs11549467 and rs41508050 polymorphism was not associated with CAD (OR = 1.16, 95% CI = 0.91–1.47, *p* = 0.22, *I*^2^ = 10%; OR = 1.94, 95% CI = 0.99–3.81, *p* = 0.05, *I*^2^ = 6%) (Additional file 1: Figures S5 and S6).

## Discussion

In the present study, we aimed to investigate whether genetic variations in genes that encode proteins expressed in smooth muscle cells (PECAM1, HIF1A) and endothelial cells (KIAA1462) influence MI risk in Han Chinese. Through a series of genetic testings of the three SNPs, we find that the rs1867624 and rs2057482 SNPs were significantly associated with lipid levels in in MI patients compared with the control group (*p* < 0.05). However, our results disclosed no association between the SNPs and susceptibility to MI (*p* > 0.05 for all). The allelic frequency of selected SNPs (rs1867624, *C* = 0.24 and 0.20; rs2057482, *T* = 0.21 and 0.20; rs3739998, *G* = 0.21 and 0.22 for cases and controls, respectively) all displayed in accordance with the one derived from the Asian populations, according to HaploReg v4.1 (rs1867624, *C* = 0.23; rs2057482, *T* = 0.20; rs3739998, *G* = 0.20.). The rationale for the selection of the three smooth muscle cells and endothelial cells-related genes investigated in this study is as follows. PECAM1 gene is found on the surface of platelets, monocytes, neutrophils, and may play a role in the inhibition of the formation of adherent junctions in endothelial cells [20]. PECAM-1 has been implicated in the maintenance of vascular barrier integrity; failure to restore barrier function contributes to the development of chronic inflammatory diseases such as atherosclerosis [21]. HIF1A, a natural antisense transcript of HIF1alpha, is overexpressed in the failing heart [22]. By modulating the stability of HIF1alpha messenger RNA, HIF1A has the capacity to regulate angiogenesis, an important component of the response of the heart to ischemia [23]. KIAA1462 is a protein-coding gene which is localized in the nucleus, cytosol, and plasma membrane. KIAA1462 was associated with coronary artery disease in European and South Asian population [18, 24, 25]. Akashi et al. identified the KIAA1462 as a novel protein localized at cell–cell junctions and concluded that the accumulation of KIAA1462 into endothelial cell–cell junctions depends on VE-cadherin-mediated cell–cell adhesion [17].

Our study showed that rs1867624 was associated with triglyceride (TG) levels but not with MI in Chinese population. In a previous study, Norata et al. showed that PECAM1 implicated in EC activation have been identified as modulated by triglyceride-rich lipoproteins, confirming their role in determining endothelial dysfunction [26]. In the current research, we were unable to observe a significant association of PECAM1 rs1867624 SNPs with MI in Han Chinese, However, there was considerable evidence that rs1867624 is associated with the risk of CAD in the European, South Asian, and African American [5]. Howson, et al. showed that the C-allele of PECAM1 is associated with reduced CAD risk, increased expression of PECAM1 in peripheral blood mononuclear cells, which is inconsistent with our findings. The differences in these studies may be explained by ethnic differences, environment, or lifestyle that also affected the development of MI. Another explanation is that the participants in the present study were first onset of MI, so that risk factor profile may differ with recurrent CAD patients. The mechanisms of how these loci contribute to susceptibility to MI are still not well understood. Further study is required to elucidate how these loci contribute to conferring susceptibility to MI and risk factors of MI.

Our study showed that rs2057482 was associated with HDL-cholesterol levels but we did not observe any association between HIF1A rs2057482 and the risk of MI, which is consistent with the studies by Alberto López-Reyes et al. [1]. To avoid false-negative results, we performed a meta-analysis by pooling 5 studies with totals of 2958 cases and 2474 controls to confirm the results. The rs2057482 variant allele exhibited no significant association with MI or CAD risks. The sensitivity analysis of rs2057482 found that the result of pooling ORs was significantly changed once Nan Guo’s study was excluded. These discrepancies may be attributable to the different genetic backgrounds of the study populations. To account for this difference, we performed the meta-analysis with each part considered separately. For the Caucasians populations, the HIF1A rs2057482 C > T polymorphism was associated with increased risk of CAD. For the Asians populations, the HIF1A rs2057482 C > T polymorphism was associated with decreased risk of CAD. Interestingly, the heterogeneity was consistent with the result of separate meta-analysis in Caucasians. The differences in these studies may be explained by Nan Guo’s study which has significant heterogeneity between the studies.

Several limitations should be acknowledged in the present study. First, the sample size was relatively small and the participants were limited to Chinese ethnicity. Second, there were differences in some clinical characteristics between the patients and controls. Although several confounders have been adjusted for the statistical analyses, we could not completely eliminate the potential influences of these factors on the results. Thirdly, age of control group may be a limitation of the study. Because cases are age-matched to controls, we cannot rule out the possibility that a proportion of controls would develop MI or CAD in the near future. Finally, the biological mechanism of genetic variants about the three genes was not conducted in this study. Larger studies should be followed up to assess the potential association of the SNPs with more complex, clinical-disease-related endpoints.

## Conclusions

The results of the present study showed that the subjects with rs1867624 CC genotype in MI cases had lower TG levels than the subjects with rs1867624 CT and rs1867624 TT genotypes. The subjects with rs2057482 CT genotype in MI patients had lower HDL-C levels than the subjects with rs2057482 CC and rs2057482 TT genotypes. Several SNPs interacted with age and sex to modify TC and CRE levels, and the risk of MI. In the meta-analysis of HIF1A, the rs11549465 C > T and rs10873142 T > C polymorphisms, but not rs2057482, rs11549467 and rs41508050 were correlated with overall MI or CAD risk. In conclusion, our research suggested a lack of contribution of the three SNPs to MI in a Chinese Han population. However, this study was designed as a pilot study and further investigations are needed to confirm our results and to elucidate unresolved questions.

## Methods

### Sample collection

A total of 401 hospitalized MI patients were enrolled at the Second Affiliated Hospital, Harbin Medical University (China) between September 2016 and November 2017. The study protocol was approved by Ethics Committee of the Second Affiliated Hospital of Harbin Medical University and all experimental procedures (DNA extraction and genotyping) complied with the 1975 Declaration of Helsinki. All participates gave written informed consent to take part in the present study. The diagnosis of MI was based on the Pakistan Risk of Myocardial Infarction Study (PROMIS) international guideline. Following screening by medically qualified research officers, patients aged 30–80 years have been eligible for inclusion as cases if they fulfill all of the following criteria: (1) sustained clinical symptoms suggestive of MI lasting longer than 20 min within the previous 24 h; (2) ECG changes of MI (i.e., new pathologic Q waves, at least 1 mm ST elevation in any two or more contiguous limb leads or a new left bundle branch block, or new persistent ST–T wave changes diagnostic of a non-Q wave MI); (3) confirmatory troponin-T measurements; and (4) no previous cardiovascular diseases, defined as self-reported history of angina, MI, coronary revascularization, transient ischemic attack, stroke, or evidence of CHD on prior ECG or in other medical records [5, 27]. A group of 409 age- (5-year bands) and sex-matched medical center patients without a history of CAD or symptoms of MI were selected as controls. Patients with cerebrovascular, neurological, or kidney disease; blood disorders; cancer; peripheral vascular disease; or autoimmune diseases were excluded from the control group. The controls were free of MI by questionnaires, history-taking, and clinical examination. The examination comprised physical examination, blood sampling, electrocardiography, chest X-ray, and Doppler echocardiography. Participant age, sex, blood pressure, lipid profile, fasting glucose, medical, drug, smoking, and alcohol histories were collected.

### SNP selection

After reviewing the literature for CAD candidate genes, there are many SNPs in the genes. To determine the investigation of loci, a tagSNP approach was used. We selected three SNPs on the basis of the following assumptions: (1) selected SNPs were established by Haploview (Broad Institute of MIT and Harvard, Cambridge, MA, USA, version 4.2) with the criteria (MAF > 0.05 and 푟2 > 0.8) from the known SNP data in the Chinese Han population (CHB + CHS); (2) SNPs information was obtained from the database, 1000 Genomes Project (http://browser.1000genomes.org); (3) SNPs were not reported in Chinese Hans; and (4) SNPs might be associated with the smooth muscle cells and endothelial cells in recent studies.

### SNP genotyping

The genomic DNA was extracted using a GeneJET Whole Blood Genomic DNA Purification Mini Kit (Thermo Scientific, USA) as per the product instruction. The SNP genotyping work was performed using an improved multiplex ligation detection reaction (iMLDR) technique developed by Genesky Biotechnologies Inc. (Shanghai, China). A multiplex PCR-ligase detection reaction method was used in the iMLDR. For each SNP, the alleles were distinguished by different fluorescent labels of allele-specific oligonucleotide probe pairs. Different SNPs were further distinguished by different extended lengths at the 3′end. Two negative controls were set: one with double-distilled water as template and the other with DNA sample without primers while keeping all other conditions the same in one plate. Duplicate tests were designed and the results were consistent. Genotyping call rate is 100%. A random sample accounting for ~ 5% (*n* = 40) of the total DNA samples was directly sequenced using Big Dye-terminator version 3.1 and an ABI3730XL automated sequencer (Applied Biosystems) to confirm the results of iMLDR.

### Selection of relevant studies

Study reports published before June 25, 2018 were retrieved from PubMed using the search terms (coronary artery disease, coronary heart disease, or myocardial infarction), (“HIF1A” or “MOP1”), and (polymorphism, variant, or mutation). Case-control studies of the relationship between HIF1A polymorphism and CAD or MI were eligible. At least two studies of HIF1A polymorphism reporting the genotype frequencies of each included HIF1A SNP (i.e., rs2057482, rs11549465, rs10873142, rs11549467, or rs41508050) were desired. Only studies published in English were eligible. Studies were excluded if they were not investigations of HIF1A SNPs (rs2057482, rs11549465, rs10873142, rs11549467, or rs41508050), were duplicate publications of the same population, or did not include a control group. Six articles including 3314 cases and 2687controls were selected (Fig. 1).

### Statistical analyses

All statistical analyses were performed using SPSS 13.0 (SPSS Inc., Chicago, IL, USA) and Microsoft Excel 2016 (Microsoft Corp., Redmond, WA, USA). All tests were two-sided and *p* values < 0.05 were considered significant. Between-group differences in demographic characteristics and genotype frequencies of the three SNPs were evaluated by Student’s *t* test for continuous variables and χ^2^ tests for categorical variables. The Hardy–Weinberg equilibrium was assessed for controls using the goodness-of-fit χ^2^ test (with 1 degree of freedom). A chi-square analysis was used to evaluate the difference in genotype distribution between 409 controls and 401 MI patients. Associations of genotypes and alleles and the risk of MI were estimated by odds ratios (ORs) and 95% confidence intervals (CIs). Differences of lipid levels and genotypes were determined by the Kruskal–Wallis test. Significant interactions of the three SNPs with alcohol consumption, cigarette smoking, age, sex, and hypertension with lipid levels and the risk of MI were detected by the independent-samples *t* test for categorical variables and linear regression analysis for continuous variables after controlling for potential confounders; a *p* value < 0.003 after the Bonferroni correction was considered statistically significant.

Revman 5.3 software (Nordic Cochrane Centre, Cochrane Collaboration, Copenhagen, Denmark) was used to for the meta-analysis. ORs and their 95% CIs were calculated to determine the significance of associations between HIF1A allele genotypes and susceptibility to MI or CAD. Heterogeneity was tested with the χ^2^-based *Q* and *I*^2^ tests. The pooled OR was calculated using a fixed effect model in the absence of heterogeneity (*p* > 0.05, *I*^2^ < 50%). Otherwise, a random effect model was used. The stability of the pooled ORs was determined by one-way sensitivity analysis.

## Additional file

Additional file 1: **Figure S1.** The sensitivity analysis of HIF1A rs2057482. **Figure S2.** The meta-analysis of HIF1A rs2057482 in Caucasians. **Figure S3.** The meta-analysis of HIF1A rs2057482 in Asian. **Figure S4.** The meta analysis of HIF1A rs10873142. **Figure S5.** The meta analysis of HIF1A rs11549467. **Figure S6.** The meta analysis of HIF1A rs41508050. (PPTX 1606 kb)

## Abbreviations

CAD: Coronary artery disease
ECs: Endothelial cells
HIF1A: Hypoxia inducible factor 1 subunit alpha
MI: Myocardial infarction
PECAM1: Platelet endothelial cell adhesion molecule 1
SMCs: Smooth muscle cells
SNP: Single-nucleotide polymorphism

## References

[1] Lopez-Reyes A, Rodriguez-Perez JM, Fernandez-Torres J, Martinez-Rodriguez N, Perez-Hernandez N, Fuentes-Gomez AJ, Aguilar-Gonzalez CA, Alvarez-Leon E, Posadas-Romero C, Villarreal-Molina T (2014). The HIF1A rs2057482 polymorphism is associated with risk of developing premature coronary artery disease and with some metabolic and cardiovascular risk factors. The genetics of atherosclerotic disease (GEA) Mexican study. Exp Mol Pathol.

[2] Lao KH, Zeng L, Xu Q (2015). Endothelial and smooth muscle cell transformation in atherosclerosis. Curr Opin Lipidol.

[3] Yurchenco PD. Basement membranes: cell scaffoldings and signaling platforms. Cold Spring Harb Perspectives Biology. 2011;3(2):a004911. 10.1101/cshperspect.a004911 PMC3039528 21421915

[4] Yang W, Ng FL, Chan K, Pu X, Poston RN, Ren M, An W, Zhang R, Wu J, Yan S (2016). Coronary-heart-disease-associated genetic variant at the COL4A1/COL4A2 locus affects COL4A1/COL4A2 expression, vascular cell survival, atherosclerotic plaque stability and risk of myocardial infarction. PLoS Genet.

[5] Howson JMM, Zhao W, Barnes DR, Ho WK, Young R, Paul DS, Waite LL, Freitag DF, Fauman EB, Salfati EL (2017). Fifteen new risk loci for coronary artery disease highlight arterial-wall-specific mechanisms. Nat Genet.

[6] Coronary Artery Disease C, Samani NJ, Deloukas P, Erdmann J, Hengstenberg C, Kuulasmaa K, McGinnis R, Schunkert H, Soranzo N, Thompson J (2009). Large scale association analysis of novel genetic loci for coronary artery disease. Arteriosclerosis, Thromb, Vasc Biol.

[7] Makinen VP, Civelek M, Meng Q, Zhang B, Zhu J, Levian C, Huan T, Segre AV, Ghosh S, Vivar J (2014). Integrative genomics reveals novel molecular pathways and gene networks for coronary artery disease. PLoS Genet.

[8] De Caterina R, Talmud PJ, Merlini PA, Foco L, Pastorino R, Altshuler D, Mauri F, Peyvandi F, Lina D, Kathiresan S (2011). Strong association of the APOA5-1131T>C gene variant and early-onset acute myocardial infarction. Atherosclerosis.

[9] Gu A, Tsark W, Holmes KV, Shively JE (2009). Role of Ceacam1 in VEGF induced vasculogenesis of murine embryonic stem cell-derived embryoid bodies in 3D culture. Exp Cell Res.

[10] Sakowicz A, Fendler W, Lelonek M, Sakowicz B, Pietrucha T (2013). Genetic polymorphisms and the risk of myocardial infarction in patients under 45 years of age. Biochem Genet.

[11] Nasibullin TR, Timasheva YR, Sadikova RI, Tuktarova IA, Erdman VV, Nikolaeva IE, Sabo J, Kruzliak P, Mustafina OE (2016). Genotype/allelic combinations as potential predictors of myocardial infarction. Mol Biol Rep.

[12] Auer J, Weber T, Berent R, Lassnig E, Lamm G, Eber B (2003). Genetic polymorphisms in cytokine and adhesion molecule genes in coronary artery disease. Am J Pharmacogenomics : genomics-related research in drug development and clinical practice.

[13] Incalcaterra E, Hoffmann E, Averna MR, Caimi G (2004). Genetic risk factors in myocardial infarction at young age. Minerva Cardioangiol.

[14] Imanishi M, Tomita S, Ishizawa K, Kihira Y, Ueno M, Izawa-Ishizawa Y, Ikeda Y, Yamano N, Tsuchiya K, Tamaki T (2014). Smooth muscle cell-specific Hif-1alpha deficiency suppresses angiotensin II-induced vascular remodelling in mice. Cardiovasc Res.

[15] Guo N, Zhang N, Yan L, Cao X, Wang J, Wang Y (2017). Correlation between genetic polymorphisms within the MAPK1/HIF-1/HO-1 signaling pathway and risk or prognosis of perimenopausal coronary artery disease. Clin Cardiol.

[16] Goncharova IA, Makeeva OA, Golubenko MV, Markov AV, Tarasenko NV, Sleptsov AA, Puzyrev VP (2016). Genes for fibrogenesis in the determination of susceptibility to myocardial infarction. Mol Biol.

[17] Akashi M, Higashi T, Masuda S, Komori T, Furuse M (2011). A coronary artery disease-associated gene product, JCAD/KIAA1462, is a novel component of endothelial cell-cell junctions. Biochem Biophys Res Commun.

[18] Erdmann J, Willenborg C, Nahrstaedt J, Preuss M, Konig IR, Baumert J, Linsel-Nitschke P, Gieger C, Tennstedt S, Belcredi P (2011). Genome-wide association study identifies a new locus for coronary artery disease on chromosome 10p11.23. Eur Heart J.

[19] Hara T, Monguchi T, Iwamoto N, Akashi M, Mori K, Oshita T, Okano M, Toh R, Irino Y, Shinohara M (2017). Targeted disruption of JCAD (junctional protein associated with coronary artery disease)/KIAA1462, a coronary artery disease-associated gene product, inhibits angiogenic processes in vitro and in vivo. Arterioscler Thromb Vasc Biol.

[20] Listi F, Caruso C, Di Carlo D, Falcone C, Boiocchi C, Cuccia M, Candore G (2010). Association between platelet endothelial cellular adhesion molecule-1 polymorphisms and atherosclerosis: results of a study on patients from northern Italy. Rejuvenation Res.

[21] Privratsky JR, Paddock CM, Florey O, Newman DK, Muller WA, Newman PJ (2011). Relative contribution of PECAM-1 adhesion and signaling to the maintenance of vascular integrity. J Cell Sci.

[22] Zolk O, Solbach TF, Eschenhagen T, Weidemann A, Fromm MF (2008). Activation of negative regulators of the hypoxia-inducible factor (HIF) pathway in human end-stage heart failure. Biochem Biophys Res Commun.

[23] Amaral N, Okonko DO (2015). Mitigation of myocardial ischemia-reperfusion injury via HIF-1alpha-frataxin signaling. Am J Phys Heart Circ Phys.

[24] Murdock DG, Bradford Y, Schnetz-Boutaud N, Mayo P, Allen MJ, D'Aoust LN, Liang X, Mitchell SL, Zuchner S, Small GW (2013). KIAA1462, a coronary artery disease associated gene, is a candidate gene for late onset Alzheimer disease in APOE carriers. PLoS One.

[25] Coronary Artery Disease Genetics C (2011). A genome-wide association study in Europeans and South Asians identifies five new loci for coronary artery disease. Nat Genet.

[26] Norata GD, Grigore L, Raselli S, Seccomandi PM, Hamsten A, Maggi FM, Eriksson P, Catapano AL (2006). Triglyceride-rich lipoproteins from hypertriglyceridemic subjects induce a pro-inflammatory response in the endothelium: molecular mechanisms and gene expression studies. J Mol Cell Cardiol.

[27] Saleheen D, Zaidi M, Rasheed A, Ahmad U, Hakeem A, Murtaza M, Kayani W, Faruqui A, Kundi A, Zaman KS (2009). The Pakistan risk of myocardial infarction study: a resource for the study of genetic, lifestyle and other determinants of myocardial infarction in South Asia. Eur J Epidemiol.

[28] Hlatky MA, Quertermous T, Boothroyd DB, Priest JR, Glassford AJ, Myers RM, Fortmann SP, Iribarren C, Tabor HK, Assimes TL (2007). Polymorphisms in hypoxia inducible factor 1 and the initial clinical presentation of coronary disease. Am Heart J.

[29] Duran J, Olavarria PS, Mola M, Gotzens V, Carballo J, Pelegrina EM, Petit M, Abdul-Jawad O, Otaegui I, del Blanco BG (2015). Genetic association study of coronary collateral circulation in patients with coronary artery disease using 22 single nucleotide polymorphisms corresponding to 10 genes involved in postischemic neovascularization. BMC Cardiovasc Disord.

[30] Zheng ZL, Hwang YH, Kim SK, Kim S, Son MJ, Ro H, Sung SA, Lee HH, Chung WK, Joo KW (2009). Genetic polymorphisms of hypoxia-inducible factor-1 alpha and cardiovascular disease in hemodialysis patients. Nephron Clin Pract.

[31] Liu Q, Liang Y, Zou P, Ni WX, Li YG, Chen SM (2013). Hypoxia-inducible factor-1alpha polymorphisms link to coronary artery collateral development and clinical presentation of coronary artery disease. Biomed Pap Med Fac Univ Palacky, Olomouc, Czech.

