# Supplementary material for: The association of functional polymorphisms in genes expressed in endothelial cells and smooth muscle cells with the myocardial infarction
Source: Hum Genomics. 2019 Jan 24;13:5. doi: 10.1186/s40246-018-0189-8 (PMC6345039; doi:10.1186/s40246-018-0189-8)
Supplement: Supplementary file 1 — Figure S1. The sensitivity analysis of HIF1A rs2057482. Figure S2. The meta-analysis of HIF1A rs2057482 in Caucasians. Figure S3. The meta-analysis of HIF1A rs2057482 in Asian. Figure S4. The meta analysis of HIF1A rs10873142. Figure S5. The meta analysis of HIF1A rs11549467. Figure S6. The meta analysis of HIF1A rs41508050. (PPTX 1606 kb) [file 40246_2018_189_MOESM1_ESM.pptx]

## Slide 1
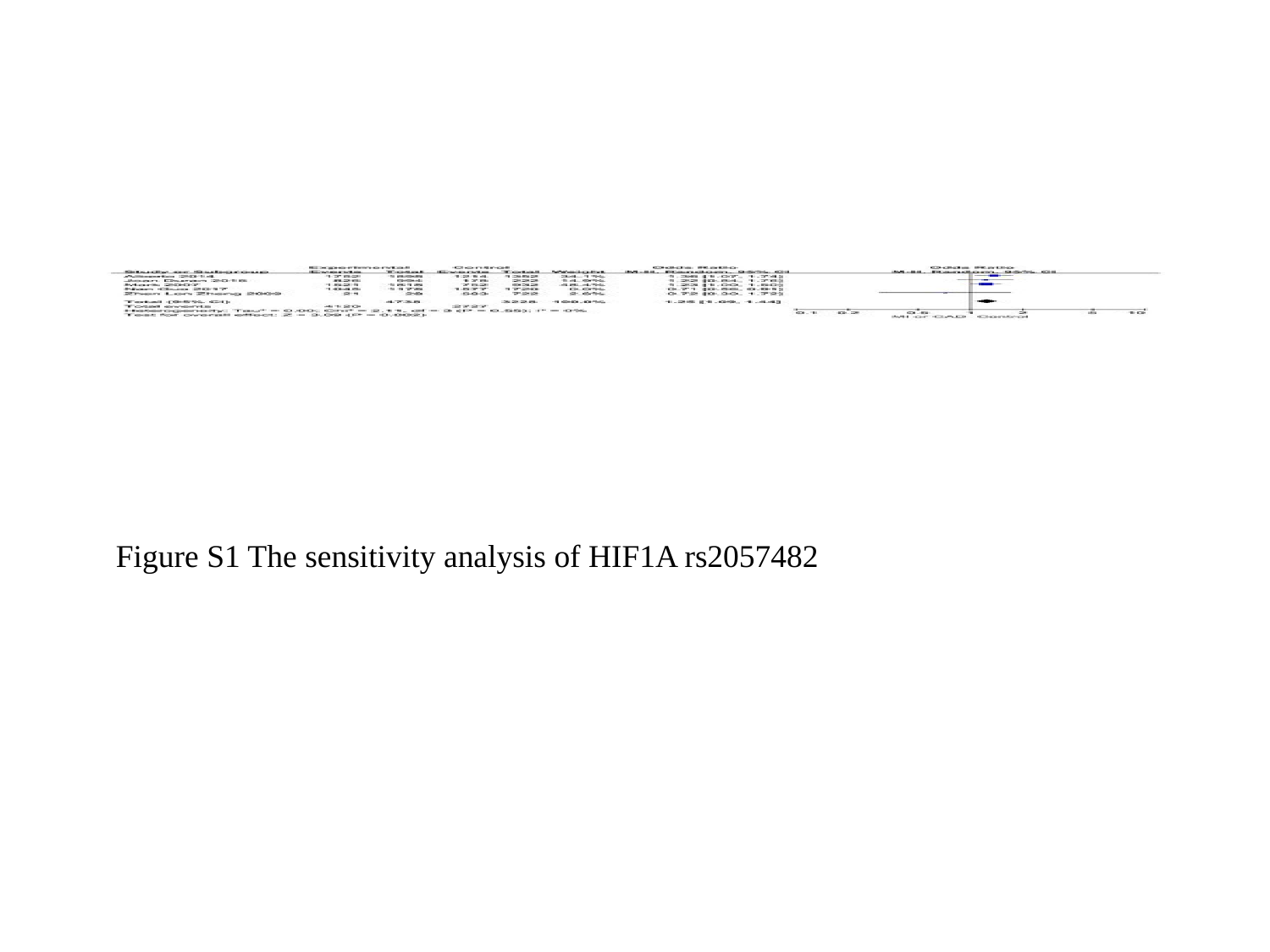

Figure S1 The sensitivity analysis of HIF1A rs2057482

## Slide 2
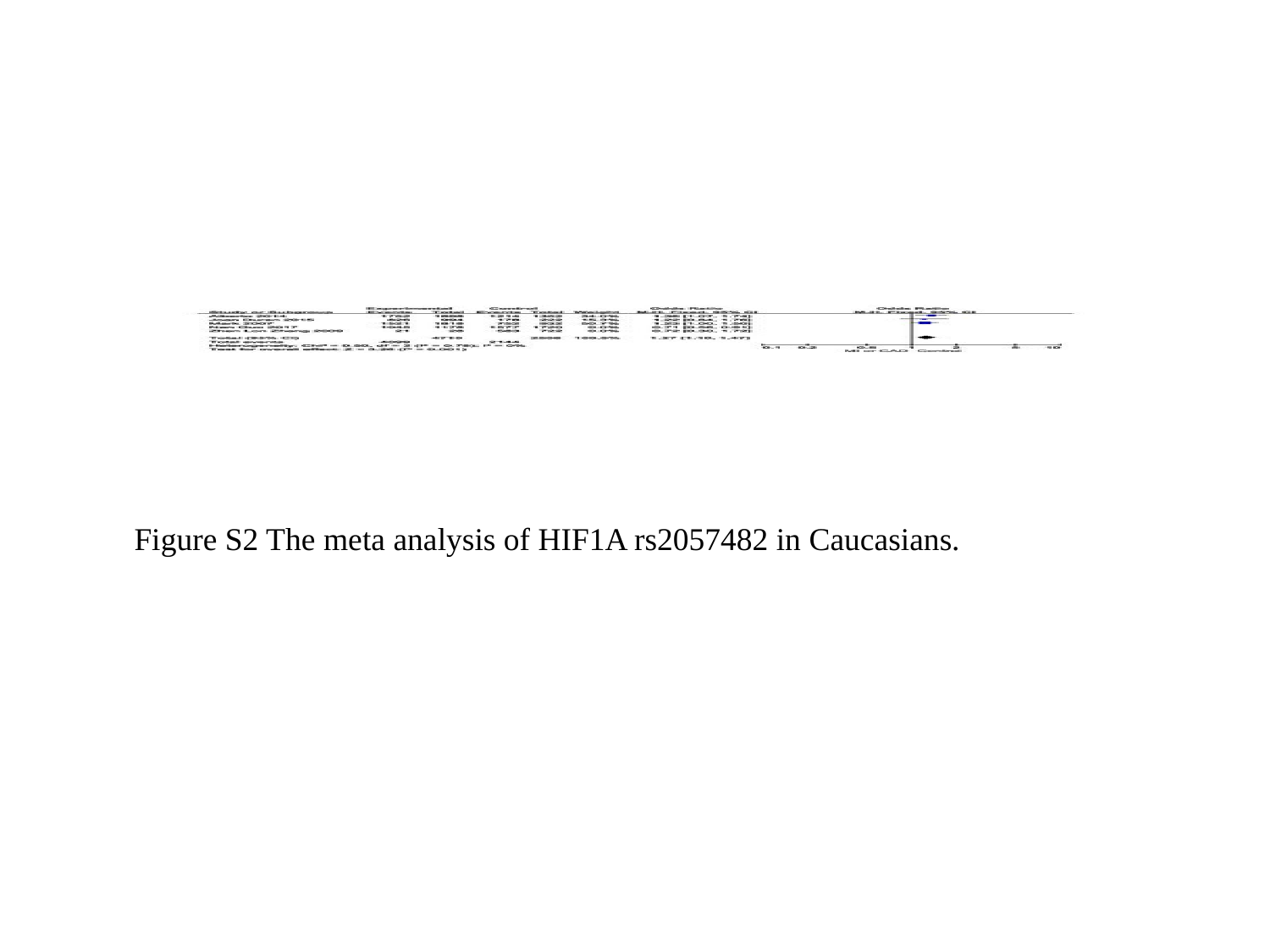

Figure S2 The meta analysis of HIF1A rs2057482 in Caucasians.

## Slide 3
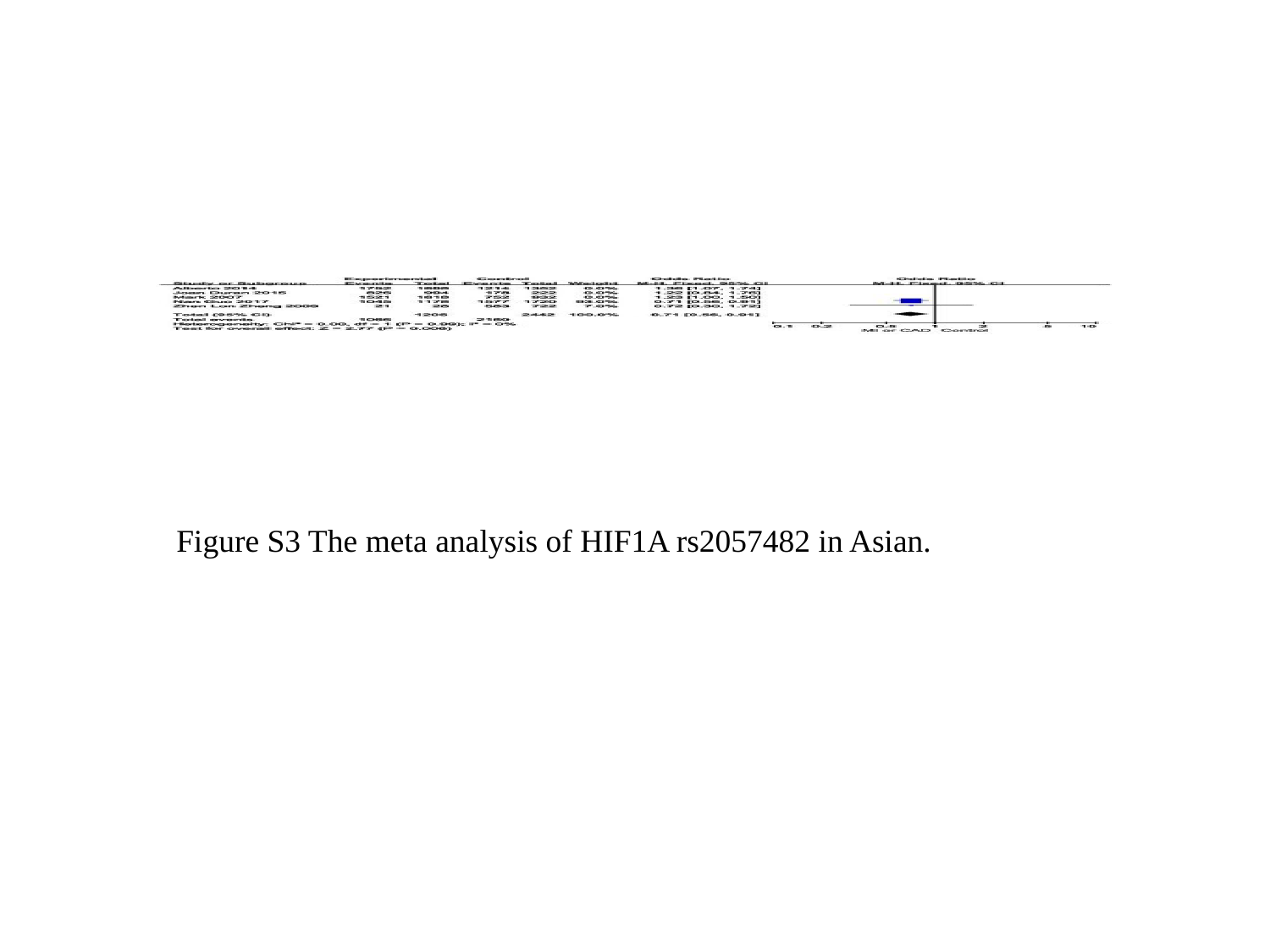

Figure S3 The meta analysis of HIF1A rs2057482 in Asian.

## Slide 4
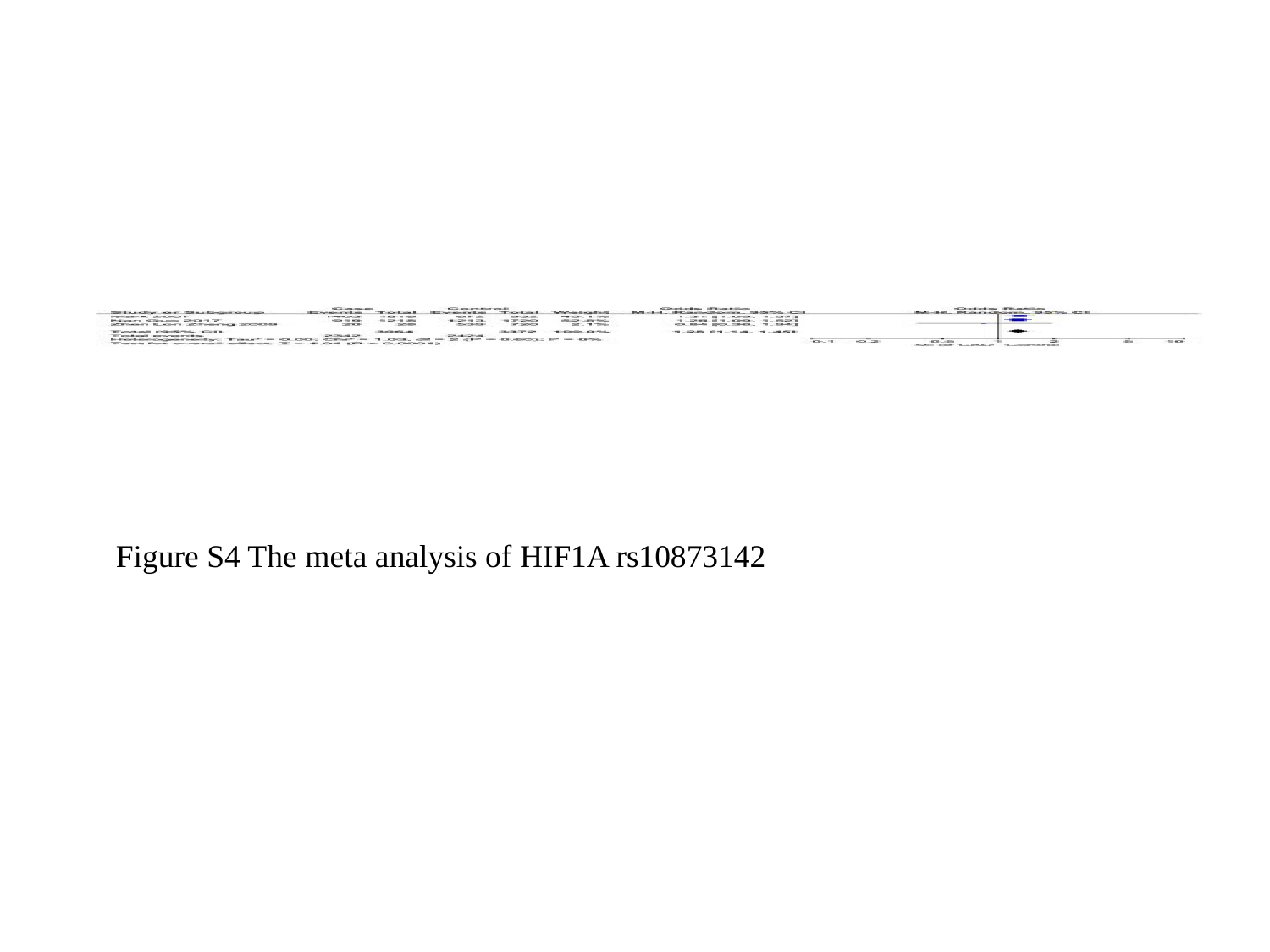

Figure S4 The meta analysis of HIF1A rs10873142

## Slide 5
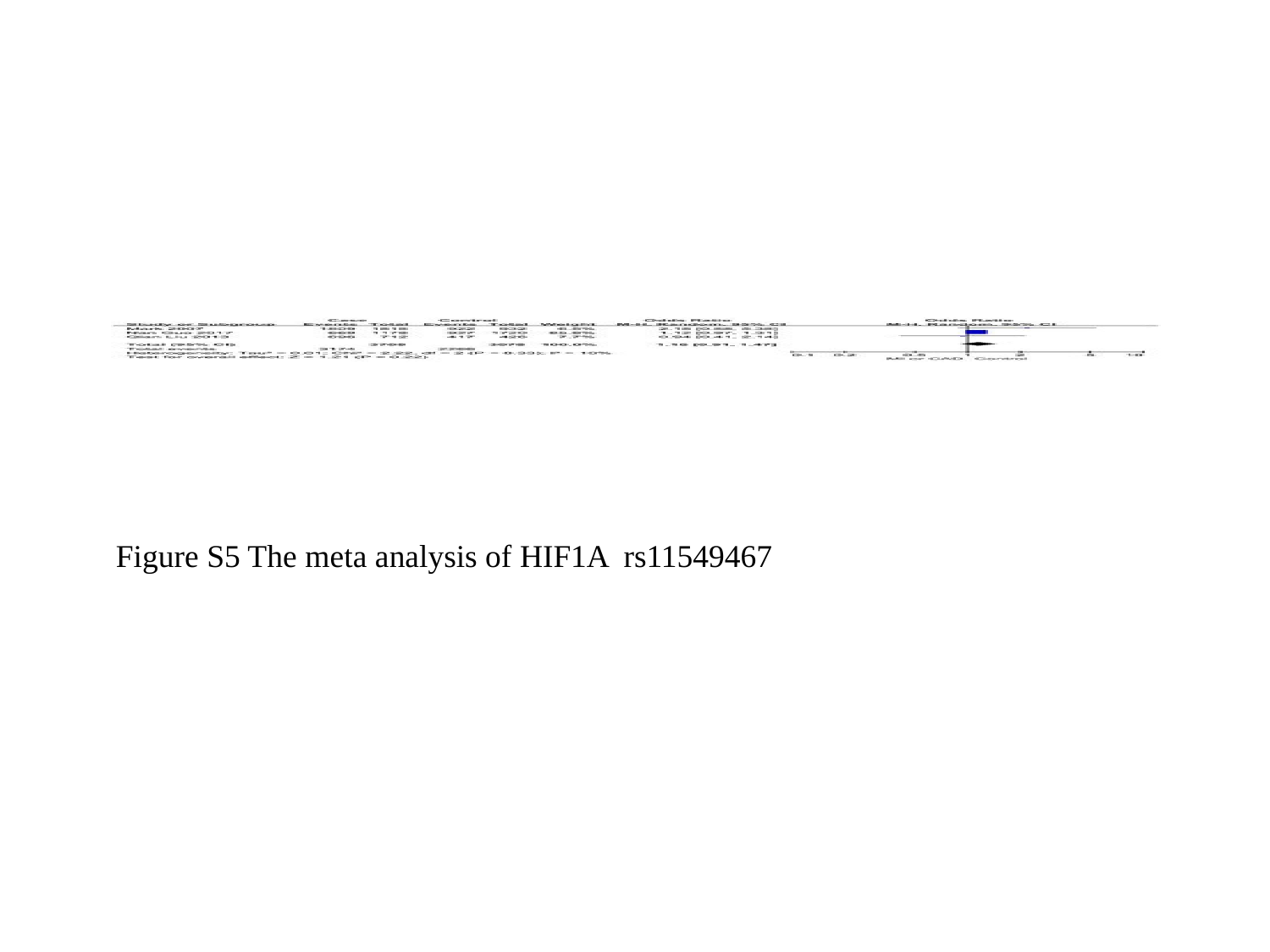

Figure S5 The meta analysis of HIF1A rs11549467

## Slide 6
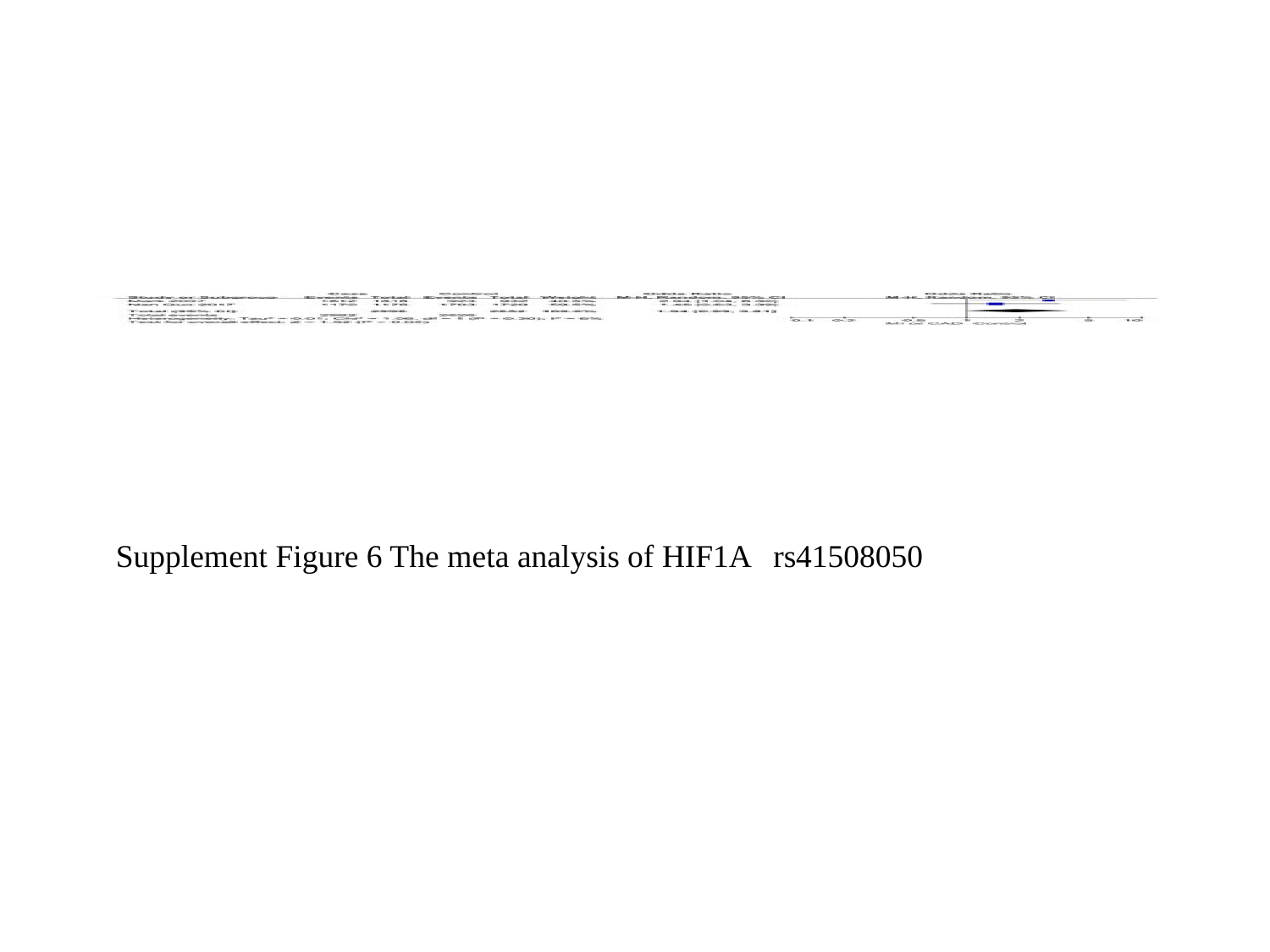

Supplement Figure 6 The meta analysis of HIF1A rs41508050
